# Botanical Mixture Containing Nitric Oxide Metabolite Enhances Neural Plasticity to Improve Cognitive Impairment in a Vascular Dementia Rat Model

**DOI:** 10.3390/nu15204381

**Published:** 2023-10-16

**Authors:** Xiaorong Zhang, Seung-Bum Yang, Lin Cheng, Koo Ho, Min-Sun Kim

**Affiliations:** 1 Department of Pathology, Affiliated Hospital of Jiujiang University, Jiujiang 332000, China; 2 Center for Cognitive Science and Transdisciplinary Studies, Jiujiang University, Jiujiang 332000, China; 3 Center for Nitric Oxide Metabolite, Wonkwang University, Iksan 54538, Republic of Korea; 4 Department of Medical Non-Commissioned Officer, Wonkwang Health Science University, Iksan 54538, Republic of Korea; 5 Jiujiang Clinical Precision Medicine Research Center, Jiujiang 332000, China

**Keywords:** vascular dementia, long-term potentiation, parvalbumin, nitric oxide, *Scutellaria baicalensis*, *Rhodiola rosea*

## Abstract

Vascular dementia (VD), caused by impaired cerebral blood flow, is the most common form of dementia after Alzheimer’s disease (AD) in the elderly and is characterized by severe neuronal damage and cognitive decline. Nitric oxide (NO) is an important determinant of vascular homeostasis, and its deficiency is associated with the progression of VD. In this study, we investigated the role of nitrite ion, a NO metabolite in a botanical mixture (BM) of fermented garlic, fermented *Scutellaria baicalensis*, and *Rhodiola rosea* on neuron loss and cognitive impairment using a VD rat model. The BM containing the NO metabolite alleviated cognitive deficits and enhanced neural plasticity, as reflected by an increase in long-term potentiation. The BM also alleviated neuron apoptosis, decreased GFAP expression, and oxidative stress, and increased parvalbumin and brain-derived neurotrophic factor (BDNF) levels. These results indicate that BM exerts neuroprotective effects and alleviates cognitive dysfunction while enhancing neuroplasticity, and thus has therapeutic potential against VD.

## 1. Introduction

Vascular dementia (VD) is the second most common form of dementia after Alzheimer’s disease (AD); it is caused by impaired cerebral blood flow. VD is characterized by severe neuronal damage, cognitive decline, white matter lesions, neurodegeneration, synapse and dendritic spine loss, and neuroinflammation, and can eventually lead to death [1,2]. There are currently no effective therapies for VD and treatments are mainly symptomatic. The prevalence of VD is increasing worldwide with the aging of the population, and there is, therefore, an urgent need for new treatment approaches.

Nitric oxide (NO) is a key signaling molecule in cell–cell communication; it is produced in cells by NO synthase (NOS) [3] and plays an important role in various biological processes in the central nervous system, including the modulation of cerebral blood flow, synaptic plasticity, neuroinflammation, oxidative stress, and apoptosis [4]. NO depletion plays an important role in VD pathogenesis [5]; thus, therapeutic strategies that increase NO levels may be beneficial in VD. Recent studies showed that various reductase can reduce the nitrite ion to NO, increasing NO bioavailability in the body. Thus, nitrite is considered as a NO metabolite [6,7].

Garlic is a plant that has medicinal value owing to its demonstrated capacity to modulate immune function [8] and reduce cardiovascular disease risk [9], and for its antidiabetic and antihypertensive properties [10]. Our previous study has demonstrated that fermented garlic, enriched in NO metabolites, increased systemic blood flow by activating intracellular nitric oxide signaling [11]. *Scutellaria baicalensis* is a flowering plant that has been widely used to treat of various neurodegenerative diseases. *S. baicalensis* and its extract have cytoprotective and anti-inflammatory effects related to the reduction in reactive oxygen species (ROS) accumulation and alleviation of mitochondrial damage [12,13]. *Rhodiola rosea*, another flowering plant, has been widely used to stimulate the nervous system and can attenuate anxiety, enhance work performance and the capacity for physical work, and improve memory and learning in rat models [14,15]. Additionally, *S. baicalensis* and *R. rosea* can increase NO production and bioavailability [16,17].

To determine whether the NO metabolite that increases NO bioavailability can be used to prevent or treat VD, the present study investigated the effects of a mixture of fermented garlic extract, fermented *S. baicalensis*, and *R. rosea* on neural plasticity and memory function in a VD rat model.

## 2. Materials and Methods

### 2.1. Animals

Adult male Sprague Dawley rats (three groups of 12 rats each, and the experiment was repeated three times) aged 7–8 weeks (Samtako Bio, Seoul, Republic of Korea), weighing 200–250 g on arrival, were housed in groups of three per cage under controlled laboratory conditions (12:12 h light/dark cycle, lights on at 7:00 a.m., 22 ± 2 °C, 45–55% humidity). The rats had ad libitum access to food and water. All experiments were approved by the Laboratory Animal Ethics Committee of Wonkwang University (WKU20-12, approval date: 23 January 2020).

### 2.2. BCCAO Surgery

Permanent bilateral occlusion of the common carotid arteries (BCCAO) in rats is used to investigate the cognitive decline and neurodegenerative processes associated with VD [18,19]. The animals were randomly divided into three groups (*n* = 12 per group): (1) sham-operated, (2) BCCAO, and (3) BCCAO + treatment with the botanical mixture (BM) containing NO metabolite (4 mL/kg administered twice a day for 4 weeks). The rats were anesthetized with an intraperitoneal injection of ketamine (5 mg/kg) and BCCAO was performed as previously described [20]. Briefly, an incision was made in the middle of the neck; the two sides of the common carotid artery and vagus nerve were carefully separated, and the common carotid artery was then permanently ligated with a silk suture (4-0). Sham-operated rats underwent the same procedure but without common carotid artery ligation.

### 2.3. BM Preparation and Administration

BM consisted of NO metabolite (2000 ppm of nitrite in 1.5 mL of diluted fermented garlic extract), ethanol extract of fermented *S. baicalensis* (1 g), and fermented *R. rosea* (1 g), both diluted in 50 mL of distilled water. The fermented garlic extract was supplied by HumanEnos (Wanju-gun, Jeonbuk, Republic of Korea). Rats in the BCCAO+BM group started receiving BM treatment on day 2 after the BCCAO surgery. The treatment lasted 4 weeks (4 mL/kg twice a day, oral administration) (10:00 a.m. and 4:00 p.m.). The rats in the sham and BCCAO groups received the same volume of saline (vehicle).

### 2.4. Y-Maze Test

**Figure 1:**
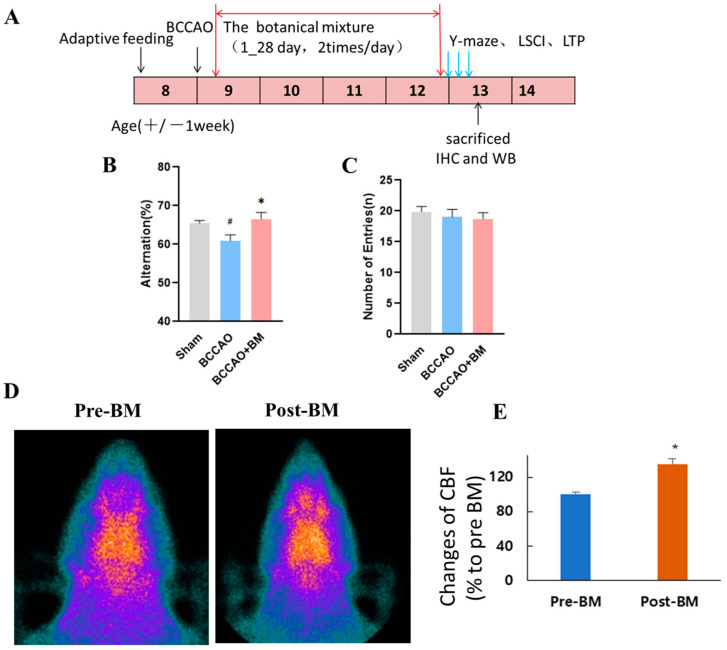
Botanical mixture alleviates cognitive deficits in a rat model of VD. (**A**) Treatment schedule for establishing the BCCAO rat model. (**B**) Performance in the Y-maze test at 4 weeks after BCCAO. (**C**) Number of arm entries in the Y-maze test. (**D**) Change in cerebral blood flow evaluated by SPECT imaging using 99mTcHMPAO tracer (*n* = 3). (**E**) Bar histogram showing changes in the cerebral blood flow (CBF) calculated by cerebral hemisphere/neck soft tissue uptake ratios before and after treatment of BM in 3 BACCO rats. Values represent percent ratio of CBF of Post-BM to that of Pre-BM and are mean ± SD (*n* = 3, * *p* < 0.05 Pre-BM vs. Post-BM). # *p* < 0.05 vs. sham; * *p* < 0.05 vs. BCCAO (Y-maze, *n* = 12 per group).

Four weeks after BCCAO surgery, the Y-maze was performed to assess spatial learning and memory (Figure 1A) [20]. All animals were trained daily at 3:00 p.m. Training times and conditions were consistent, and all the training was carried out by the same researcher. The Y-maze consisted of three arms (A, B, and C) evenly spaced at a 120° angle. The animals were placed in the center of the Y-maze and allowed to freely explore for 8 min. The sequence of entries into each arm by each rat was recorded by a video tracking and analysis system.

### 2.5. Brain-Perfusion SPECT Imaging

Tc-99m ECD (~185 MBq) was injected intravenously into the rats. After 30 min, brain images were acquired using a gamma camera equipped with a 3 mm pinhole collimator (Vertex; ADAC Laboratories, Milpitas, CA, USA) with the following settings: window setting at 140 keV, width of 20%, and acquisition time of 150 s. The images were saved in a 512 × 512 matrix. Regions of interest were manually drawn in the cerebral hemisphere, brain stem, and neck soft tissue areas for analysis. The cerebral hemisphere/brain stem (Cb/bs) and cerebral hemisphere/neck soft tissue (Cb/neck) uptake ratios were calculated using the mean radioactivity of the brain stem and neck soft tissue as a reference.

Brain perfusion images were obtained from three animals four weeks after BCCAO. Following general anesthesia using 3% isoflurane, a baseline brain perfusion image was acquired. Three days later, the rats with BCCAO were re-anesthetized and orally administered a 1 mL injection of BM mixture. After the treatment, brain perfusion images were obtained using the same method as described above. The percentage differences of Cb/bs and Cb/neck before and after treatment were calculated. 

### 2.6. Long-Term Potentiation (LTP) in the Hippocampus

Animals were anesthetized with urethane (12 mg/kg) and their head was fixed on a stereotaxis system. The skull and dura were removed, and a bipolar metal stimulation electrode was inserted into the CA3 area of the hippocampus (anterior–posterior [AP], 4.0 mm; dorsal–ventral [DV], 3.5 mm; medial–lateral [ML], 3.5 mm from bregma). A tungsten recording electrode was used to record field-evoked excitatory postsynaptic potentials (fEPSPs) in the CA1 area (AP, −3.0 mm; ML, 1.5–2.5 mm; DV, 2.6 mm). The change in fEPSP amplitude as stimulus intensity gradually increased from 0.01 to 0.2 mA was recorded, and the stimulus intensity eliciting the maximum fEPSP amplitude and 50% of the maximum amplitude was determined.

To induce LTP, electric stimulation was delivered in a theta-burst stimulation (TBS) pattern in the CA3 region three times at 1 min intervals. TBS consisted of a repetitive burst waveform (five pulses, 100 Hz) composed of five square waves at 10-ms intervals, delivered five times at 200-ms intervals (5 Hz). After TBS, fEPSPs were induced and recorded for 60 min in the same manner. After the recording, a 0.3-mA positive current was delivered via the recording electrode for 20 s to create a lesion in the CA1 and CA3 regions of the hippocampus. The location of the stimulation and recording electrodes was confirmed by Cresyl Violet staining. Signal 2.0 (Cambridge Electronic Design, Cambridge, UK) and Excel 2013 (Microsoft, Redmond, WA, USA) software programs were used to analyze fEPSP signals.

### 2.7. Immunohistochemistry

Rats were anesthetized with urethane (12 mg/kg), then transcardially perfused with 1% paraformaldehyde (PFA) and 4% PFA dissolved in a 0.2 M phosphate buffer and decapitated. The brain was removed carefully and quickly fixed in 4% PFA overnight at 4 °C, and immersed in a 30% sucrose solution for 3 days at 4 °C. Coronal brain sections (35-µm thick) were cut using a freezing microtome and incubated for 4 min at 95 °C with an antigen retrieval solution. After being washed with phosphate-buffered saline (PBS), the sections were rinsed with 0.5% PBS and 0.1% Triton X-100 containing 3% goat serum, followed by overnight incubation at 4 °C with primary antibodies against nitrotyrosine (Millipore, Billerica, MA, USA; cat. no. 06-284, 1:300), parvalbumin (PV) (Abcam, Cambridge, MA, USA; cat. no. ab11427, 1:2000), and neuronal nuclei (NeuN) (Abcam; cat. no. ab128886, 1:1000). They were then washed with 0.05% PBS and incubation for 1 h with horseradish peroxidase (HRP)-conjugated anti-rabbit IgG (Golden Bridge International Labs, Uden, The Netherlands; cat. no. D13-110). Immunoreactivity was visualized with 0.05% diaminobenzidine (DAB) in hydrochloric acid and 0.003% H_2_O_2_. The tissue was washed with 0.1 M phosphate buffer, mounted on gel-coated slides, dehydrated, cleared with xylene, and mounted on slides; microscopy pictures were then recorded. Three sections per brain and three fields per section were used for quantification and the ImageJ software (1.8.0) was used to calculate the total number or strength of positive cells in each field.

### 2.8. Cresyl Violet Staining

The rat brain sections were rinsed for 2 min with 100% alcohol and xylene for 2 min, followed by 100%, 70%, and 20% alcohol for 2 min each. The sections were then incubated in a 0.1% Cresyl Violet solution for 5 min, then washed with distilled water, differentiated in 95% alcohol for 1 min, dehydrated in 100% alcohol for 2 min, cleared in xylene, and mounted using a DPX mounting medium.

### 2.9. Western Blot Analysis

Cortex and hippocampus tissues were homogenized in a buffer containing 1× inhibitor cocktail and 0.5 mM EDTA solution. Total proteins were quantified with a BCA Protein Assay Kit, and a sample of proteins (20 μg) was separated on a 10%, 12%, or 15% sodium dodecyl sulfate (SDS)-polyacrylamide gel for 90 min at 100 V and transferred to an Immobilon P membrane that was blocked with 5% dried milk protein for 1 h, rinsed three times with 0.05% Tween-20 (TBST), and incubated with primary antibodies against PV, BDNF (Abcam; cat. no. ab108319), β-actin (Calbiochem, San Diego, CA, USA; cat. no. D00024369), and glial fibrillary acidic protein (GFAP) (Abcam; cat. no. ab7260) overnight at 4 °C. The membrane was washed three times with TBST for 10 min and incubated with HRP-conjugated secondary antibody anti-rabbit (1:5000, Santa Cruz Biotechnology, Santa Cruz, CA, USA; cat. no. sc-2357) or anti-mouse IgG (1:5000, Cell Signaling Technology, Danvers, MA, USA, cat. no. 7076S,) at room temperature for 1 h. The membrane was washed three times with TBST for 10 min and protein bands were visualized with enhanced chemiluminescent substrate (Thermo Fisher Scientific, Waltham, MA, USA, cat. no. 34577). Protein levels were quantified with ImageJ software and the ratios of nitrated proteins, BDNF, and PV/β-actin were calculated.

### 2.10. Fluoro-Jade B (FJB) Staining

The rat brain sections were washed in distilled water for 10 min and then incubated in an 80% alcohol solution containing 1% sodium hydroxide. The sections were transferred to a 70% alcohol for 2 min. Then, the sections were incubated with a 0.06% potassium permanganate solution for 10 min. Next, the sections were rinsed in distilled water for 2 min, incubated in 0.01% FJB (Histo-Chem, Jefferson, AR, USA) with 0.1% acetic acid at room temperature for 15 min, dehydrated, and mounted with DPX and glass coverslips. The brain slide was examined by fluorescence microscopy. For the quantification of FJB+ neurons, two researchers calculated the number of FJB+ neurons in each randomized microscopic field of PFC (200X) independently from each rat by using ImageJ and the results were the average values of numbers of each rat.

### 2.11. Statistical Analysis

Data analysis was performed using Prism v8.0. Values obtained from behavioral experiments were expressed as mean ± standard error, and other values were all presented as mean ± standard deviation. Y-maze data were analyzed by the Kruskal–Wallis test, and the animal experiments were performed at least three times. *p* values < 0.05 were considered statistically significant.

## 3. Results

### 3.1. BM Alleviates Cognitive Deficits and Increases Cerebral Blood Flow in Rats with BCCAO

The cognition and memory ability of rats were tested with the Y-maze test four weeks after surgery. The rate of spontaneous alternations was lower in the BCCAO group than in the sham group (*p* < 0.05). However, BCCAO+BM rats showed a significantly higher rate of spontaneous alternations than the BCCAO rats (*p* < 0.05), indicating an improvement in short-term memory. There was no significant difference in performance between the BCCAO+BM and sham groups (Figure 1B). The number of maze arm entries did not differ significantly between the three groups (*p* > 0.05; Figure 1C), indicating that there was no impairment of motor function and that BM specifically improved cognitive function and memory in BCCAO rats. SPECT images obtained using the 99mTc-HMPAO tracer revealed that BM increased cerebral blood flow after surgery (Figure 1D,E).

### 3.2. BM Improves LTP and Increases BDNF Expression

**Figure 2:**
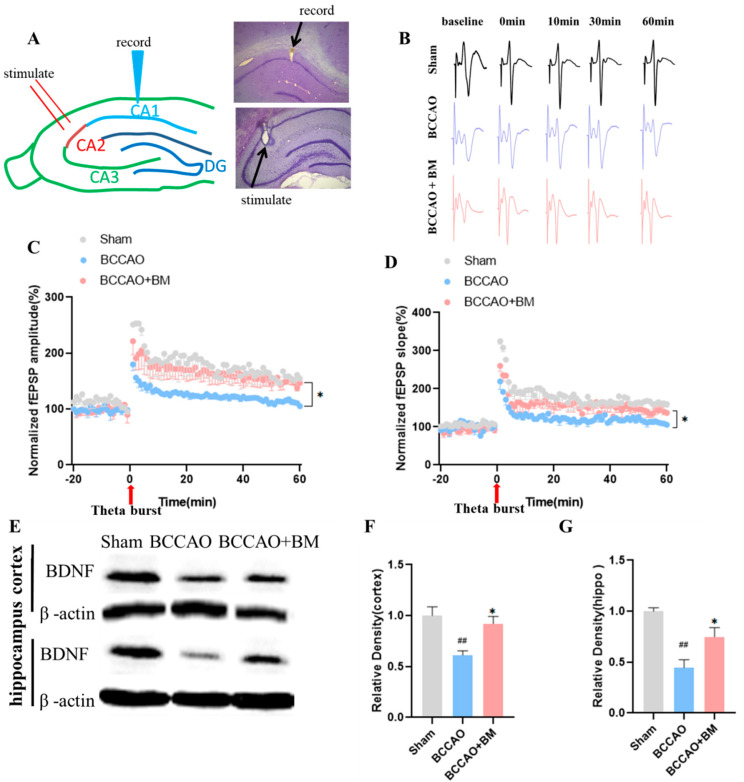
Botanical mixture improves LTP and increases BDNF expression in rats with BCCAO. (**A**) Schematic illustration and location of the stimulation site for LTP and recording site in the hippocampus CA1 area. (**B**) Representative fEPSP traces. (**C**,**D**) Time-dependent changes in amplitude and slope of fEPSP potentiation in the hippocampus CA1 area following LTP induction. (**E**) BDNF and β-actin expression in the PFC and hippocampus. (**F**,**G**) Quantification of BDNF protein levels in the PFC (**F**) and hippocampus (**G**). Values are mean ± SEM of normalized amplitudes or slope of fEPSPs (one-way analysis of variance: amplitude, F [2, 237] = 5, *p* < 0.0001; slope: F [2, 237] = 3, *p* < 0.0001). ## *p* < 0.01 vs. sham; * *p* < 0.05, vs. BCCAO (*n* = 6 per group [LTP] and 4 [BDNF]).

To investigate the effect of BM on the BCCAO-induced inhibition of LTP at the CA3–CA1 synapse, we analyzed the slope of fEPSPs, a marker of short-term synaptic plasticity. Evoked synaptic responses were assessed by calculating the average slope of fEPSPs 5 to 60 min after TBS. High-frequency stimulation-induced robust LTP in the sham and BCCAO+BM groups (Figure 2A,B). The amplitude of fEPSPs increased by 175.45% at 30 min and by 154.65% 60 min after TBS in the sham group; this was significantly greater than the increases observed in the BCCAO group (125.0% and 105.2%, respectively; F [2, 237] = 5, *p* < 0.0001; Figure 2C). Meanwhile, BM treatment reversed the BCCAO-induced suppression of LTP (*p* = 0.017; Figure 2C), with fEPSP amplitudes of 163.44% at 30 min and 146.70% at 60 min after TBS. Similar trends were observed in the slope of fEPSPs after TBS, which was significantly lower in the BCCAO group than in the sham group (*p* < 0.0001; Figure 2D) and restored by BM treatment (*p* = 0.017; Figure 2D). These data indicate that BCCAO inhibited LTP in the hippocampus and that the effect was partially reversed by treatment with BM.

BDNF is involved in neuroplasticity and may directly regulate the structure and number of synapses [21]. BDNF protein levels were significantly lower in the prefrontal cortex (PFC) (*p* = 0.0034; Figure 2E,F) and hippocampus (*p* = 0.0011; Figure 2E,G) of BCCAO rats than in sham rats, as determined by Western blotting. However, BM reversed this decrease to some extent (*p* < 0.05; Figure 2E–G).

### 3.3. BM Alleviates Damage to PV+ Neurons and Neuron Death in the Cortex and Hippocampus of BCCAO Rats

**Figure 3:**
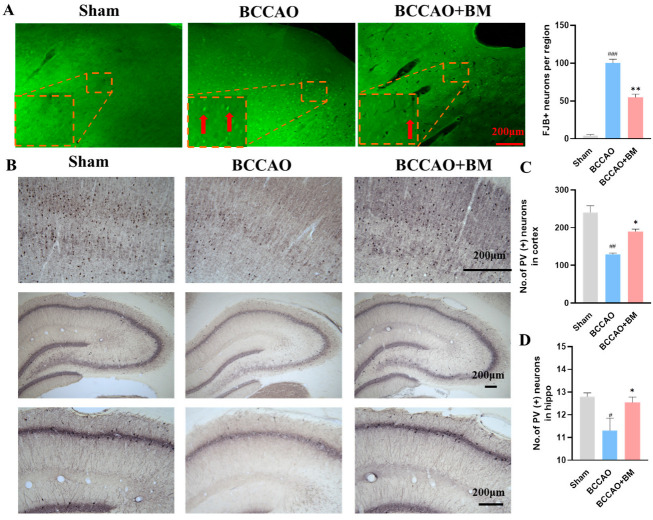

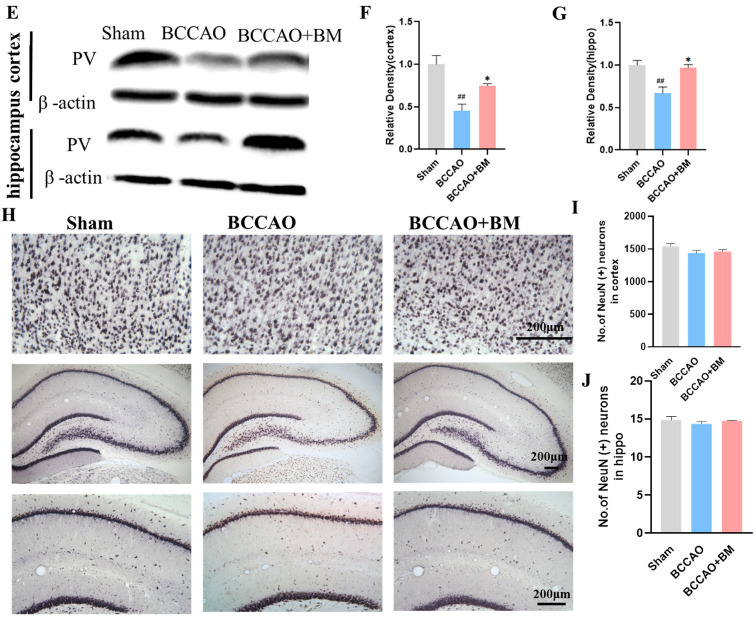
Botanical mixture administration alleviates neuronal damage and death in the PFC and hippocampus of rats with BCCAO. (**A**) FJB staining in the PFC (FJB+ neurons were showed with red arrow) and the quantification of FJB+ neurons in each group. Scale bar, 200 μm. (**B**) PV immunolabeling in the PFC and hippocampus. (**C**,**D**) Quantitative analysis of the PV-positive neurons in the PFC (**C**) and the whole hippocampus (**D**). Scale bar, 200 μm. (**E**) PV and β-actin expression in the PFC and hippocampus. (**F**,**G**) Quantification of PV protein levels in the PFC (**F**) and hippocampus (**G**). (**H**) NeuN immunolabeling in the PFC and hippocampus. Scale bar, 200 μm. (**I**,**J**) Quantification of NeuN protein levels in the PFC and the whole hippocampus. # *p* < 0.05, ## *p* < 0.01, ### *p* < 0.001 vs. sham; * *p* < 0.05, ** *p* < 0.01 vs. BCCAO (*n* = 4 per group).

The neuroprotective effect of BM in the PFC and hippocampus was examined by FJB staining and immunohistochemistry. There were more FJB+ neurons in the PFC of the BCCAO rats than in the sham group (Figure 3A), indicating that BCCAO increased neuron death. In contrast, there were fewer FJB+ neurons in BCCAO+BM rats than in BCCAO rats. 

The Ca^2+^-binding protein PV is highly expressed in gamma-aminobutyric acid (GABA)ergic interneurons [22] and modulates cognitive function. The sham group had many PV+ neurons in the PFC and hippocampus, and these had an orderly arrangement with clear nuclei (Figure 3A). However, the number of PV+ neurons and dendritic spines in the PFC and hippocampus was significantly lower in the BCCAO group (*p* < 0.01; Figure 3B–D). BM treatment restored the number of PV+ neurons relative to the BCCAO group. The results obtained by immunohistochemistry were confirmed by Western blotting; PV protein levels were lower in the PFC (*p* < 0.001) and hippocampus (*p* < 0.01) of BCCAO rats than those in sham rats, but this effect was abrogated (*p* < 0.05) in BCCAO+BM rats (Figure 3E–G). These data indicate that BM reduced damage to PV+ neurons and increased PV expression, suggesting a protective effect on GABAergic interneurons. Immunohistochemical detection of NeuN revealed that there was no significant differences in the expression of NeuN+ neurons in the PFC and hippocampus across groups (*p* > 0.05; Figure 3H–J).

### 3.4. BM Reduces Oxidative Stress and GFAP Expression

**Figure 4:**
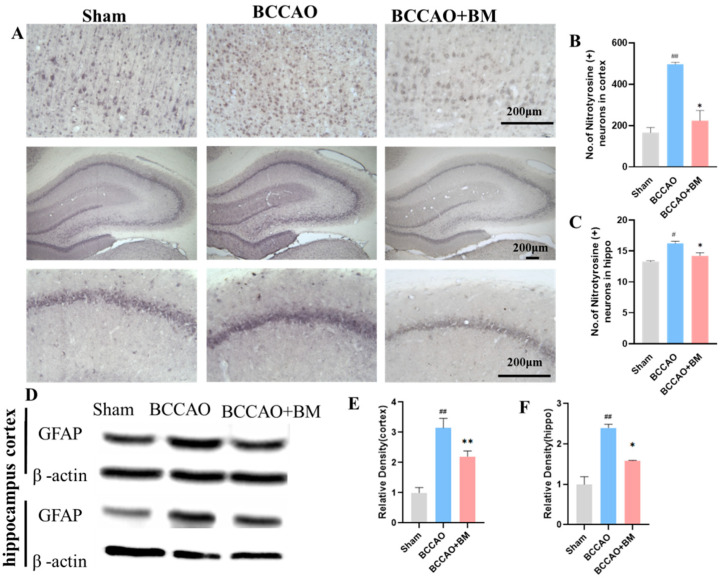
Botanical mixture alleviates oxidative stress and reduces GFAP expression. (**A**) Nitrotyrosine immunolabeling in the PFC and hippocampus. Scale bar, 200 μm. (**B**,**C**) Quantification of nitrotyrosine levels (strength of nitrotyrosine positive cells) in the PFC and hippocampus. (**D**) GFAP and β-actin expression in the PFC and hippocampus. (**E**,**F**) Quantification of the GFAP levels in the PFC (**E**) and hippocampus (**F**). # *p* < 0.05, ## *p* < 0.01 vs. sham; * *p* < 0.05, ** *p* < 0.01 vs. BCCAO (*n* = 4 per group).

VD is associated with a reduction in antioxidant function and an increase in the production of ROS-related oxidative markers [23,24]. As nitrotyrosine is a marker of oxidative stress [25], we quantified its expression immunohistochemically. The number of neurons with nitrotyrosine immunoreactivity in the PFC and hippocampus was significantly higher in the BCCAO group than in the sham group (*p* < 0.01; Figure 4A,C) but was reduced by BM treatment (*p* = 0.0106 for the PFC and *p* = 0.0364 for the hippocampus). These results indicate that BM protects neurons by reducing injury caused by oxidative stress.

GFAP is a marker of activated astrocytes that are upregulated in VD [26]. Accordingly, GFAP expression was significantly higher in the PFC (F [2, 9] = 84.25, *p* < 0.001; Figure 4D,E) and hippocampus (F [2, 9] = 33.02, *p* < 0.001; Figure 4F) of BCCAO rats than in sham rats, but BM lowered this increase (*p* < 0.01).

## 4. Discussion

The results of the present study demonstrate that the BM composed of garlic containing NO metabolite, fermented *S. baicalensis*, and *R. rosea* reduced neuron damage and death, increased cerebral blood flow, enhanced BDNF expression, reduced GFAP expression, and alleviated cognitive deficits in a rat model of VD. Moreover, BM reduced the loss of PV GABAergic interneurons caused by BCCAO and enhanced LTP, which may underlie the improvement in cognitive performance observed with BM treatment.

BCCAO in rats has been widely used to investigate chronic cerebral hypoperfusion associated with VD. Blood flow in the cortex of BCCAO rats is reduced by 42–50% in the three days post-surgery and remains at just 66% of the normal blood flow rate after four or more weeks [27,28]. Another study indicated that the blood flow in the hippocampal CA1 area was reduced from 78.4% on day 3 to 66% on week 4 [27]. Thus, the CA1 area of the hippocampus is more sensitive to ischemia than the cerebral cortex [29]. Using SPECT imaging, we found that BM increased blood flow in the brain of BCCAO rats; it also restored spatial working memory.

NO is an important multifunctional messenger molecule in the brain that plays a key role in learning and memory. NO has neuroprotective and cognition-enhancing effects and has therapeutic potential for the treatment of neurodegenerative diseases [30,31]. Fermented garlic extract contains NO metabolites such as nitrite and is considered an important medicinal plant owing to its immunomodulatory, antioxidant, antimicrobial, antidiabetic, anti-inflammatory, and antihypertensive activities [32,33,34]. Garlic also contains polyphenols and organosulfur compounds that destroy free radicals and suppress ROS generation to protect against neuronal injury [35,36]. *S. baicalensis* has anti-inflammatory, antidiabetic, antibacterial, anti-allergic, antiviral, and antihypertensive effects, and has been widely used in Oriental medicine for the treatment of brain diseases [37,38]. Baicalein, baicalin, oroxylin, and wogonin are pharmacologically active substances in *S. baicalensis* that have a strong antioxidant activity and inhibit oxidative stress caused by ROS, thereby suppressing inflammation and preventing ischemic cell damage [39]. *S. baicalensis* was shown to protect neurons from oxidative stress and reduce brain damage in ischemic brain injury models [40]; it also promotes the expression of BDNF, which is involved in neuroplasticity [41,42]. *R. rosea* has been used to alleviate anxiety and prevent high-altitude sickness and was reported to improve learning and memory and increase neurotransmitter levels, with demonstrated antioxidant activity [14,43,44]. Many of the above effects were observed in the present study in BCCAO rats treated with the combination of the three substances.

VD is characterized by an increase in ROS production, neuronal damage, activation of microglia and astrogliosis, memory deficits, and behavioral and mood changes [45,46,47]. Hippocampal LTP has been used as an electrophysiologic indicator of the formation and maintenance of memory at the synaptic level [48]. LTP was decreased to 40–77% of the baseline level in the CA1 area of the hippocampus of BCCAO rats compared with sham-operated rats four weeks after surgery [49,50]. Additionally, 8 and 16 weeks after BCCAO, the degree of LTP reduction in the CA1 region was similar to that observed in the early stage after BCCAO [50]. We found a similar degree of LTP reduction in the CA1 area four weeks post-surgery; additionally, LTP was greater in the BCCAO+BM group than in the BCCAO group. This is likely related to the observed increase in BDNF—which plays a critical role in hippocampal CA1-related memory and LTP development [51,52,53]—induced by BM treatment in the present study.

Intracellular ATP concentration is closely related to neuron death and rapidly decreases from the first three days and up to eight weeks after BCCAO [18,46,47]. Thus, damage to neurons begins in the early stages of BCCAO due to a decrease in blood flow and ATP concentration. In our study, the loss of neurons in BCCAO rats was demonstrated by FJB staining, but there were fewer FJB+ neurons in the BCCAO+BM group than in the BCCAO group. As BCCAO did not alter the number of NeuN+ neurons, we concluded that most of the FJB+ neurons were PV+ GABAergic interneurons. PV is a Ca^2+^-binding protein known to protect neurons from Ca^2+^-mediated cell death by accelerating Ca^2+^ removal after neuronal injury [54]. AD patients have reduced PV+ interneuron counts, which was related to memory decline [55,56]. BDNF selectively regulates PV expression and stimulates dendrite growth in a subset of GABAergic interneurons by activating the phospholipase C gamma pathway [57]. We found that the expression levels of PV and BDNF were significantly decreased in BCCAO rats, but this was reversed by BM treatment. Our results suggest that PV+ neurons are highly sensitive to BCCAO.

Besides neuron loss and decreased synaptic plasticity, the activation of microglia and astrocytes is related to memory and cognitive deficits in VD. Oxidative stress is a major feature of VD [38], and inflammatory cytokines have been linked to neuronal dysfunction and cognitive decline [58]. Nitrotyrosine is a marker for oxidative stress, cell damage, and inflammation that induces the activation of microglia and astrocytes [25]. In this study, nitrotyrosine levels were significantly increased in the BCCAO group but were reduced by BM treatment.

A limitation of our study is that the molecular mechanism and signaling pathways underlying the effects of BM were not investigated. In the future, a BCCAO rat model in which the synthesis of endogenous NO can be inhibited by the NOS inhibitor-l-NAME can be established to explore this question.

## 5. Conclusions

NO metabolites in BM composed of fermented garlic extract, fermented *S. baicalensis*, and *R. rosea* alleviated cerebral hypoperfusion injury in a BCCAO rat model. This was accompanied by enhanced LTP induction, increased cerebral blood flow, the upregulation of the neuroplasticity-related factor BDNF as well as PV, and decreased GFAP expression and neuron death in the hippocampus, with a corresponding improvement in spatial memory. These results suggest that BM can directly enhance neuroplasticity in the brain by improving blood flow and exerting an antioxidant effect, highlighting its therapeutic potential for the treatment of VD.

## Data Availability

Not applicable.
